# A case study of the use of a special interest group to enhance interest in public health among undergraduate health science students

**DOI:** 10.1186/s40985-018-0089-4

**Published:** 2018-05-01

**Authors:** Arauna Louw, Astrid Turner, Liz Wolvaardt

**Affiliations:** School of Health Systems and Public Health, Faculty of Health Sciences, University of Pretoria, Private Bag X323, Pretoria, 0001 South Africa

**Keywords:** Special interest group, Undergraduate health students, Public health, Action research

## Abstract

**Background:**

Education and training of undergraduate health science students in public health are insufficient in many parts of the world. This lack is a risk as early interest in specialist training options is a predictor of future training choices. A special interest group (SIG) is one mechanism to engage students, increase awareness and generate interest in public health. The purpose of this case study was to create and study such a group at an African university.

**Case presentation:**

An action research study design was used to create and study the SIG. All interested students were invited to participate in the SIG and in the data collection procedures. Data were collected via paper-based and online questionnaires. Records of activities were documented, and a reflective diary was kept by the researcher. Seven SIG meetings were held which were less than planned—some sessions were cancelled due to general student unrest. The composition of the SIG fluctuated, but the core group of 16 students consisted of 12 females (75%) and 4 males (25%). Despite faculty-wide marketing, all the participants were medical students. The most successful marketing strategy was done by two lecturers. A total of 12 participants’ motivation (75%) was to learn more about public health. Despite the range of participants being over 4-year groups with varying schedules and commitments, a convenient day and meeting time were identified. The social capital of lecturers was harnessed to invite external guest lecturers as planned field trips proved impractical. At the mid-year point, six students (38%) thought that they would consider public health as a career choice. A decision was made to recruit new members via a seminar, and 37 possible new members were identified in the process.

**Conclusions:**

A SIG appears to be an effective strategy to increase public health interest among students. This finding is key in settings with particular health workforce shortages and high burdens of disease. A foundation phase with high levels of academic support by those already qualified is needed to allow student leadership to emerge. Despite the modified and reduced number of sessions, the SIG was still successful in increasing awareness about public health and possible career choices: both positive consequences of engaging with students within a SIG.

## Background

Public health has multiple interpretations. On the one hand, some consider public health as government-funded or public sector health services [1], while others might regard it as ‘community health’ services rendered by clinical medicine [2]. In this study, public health is regarded as the field of study that focuses on the maintenance and promotion of health at a population level [3].

Public health has emerged as an important field of study given the global pandemics, poor maternal and child health outcomes and the presence of concurrent infectious and chronic diseases [4]. Despite its acknowledged importance, public health is often an unpopular inclusion in the medical curriculum and this lack of popularity is compounded by a poor understanding of public health and an opinion that public health is irrelevant to clinical work [5].

Medical students need to graduate with a basic understanding about public health and the social determinants of health in order to be able to practice medicine in a society where the growing burden of non-communicable and communicable diseases requires health promotion and disease prevention competence [5].

### Profile of South Africa’s health workforce challenges

South Africa (SA) has a quadruple burden of disease and substantial health workforce challenges that limit the country’s ability to adequately meet the population’s health needs. SA has significantly fewer doctors, nurses, pharmacists and oral health practitioners per 10,000 population than similar countries. For example, Brazil has 17.31 doctors and 65.59 nurses per 10,000 population, while SA only has 5.43 doctors and 36.1 nurses per 10,000 population. These workforce shortfalls impact on the health of the population: the SA infant mortality rate is 43.1 per 1000 live births compared to 17.3 per 1000 live births in Brazil. Similarly, the maternal mortality ratio (MMR) in SA is 165.5 per 100,000 live births, compared to the far better MMR of 75 per 100,000 live births in Brazil [6].

The structure of the South African health system is partially to blame for some of the health workforce challenges as there is a publicly funded sector that is characterised by a large client population and disproportional small health workforce and a privately funded sector with a small client population and a disproportional large health workforce [7].

South Africa has adopted a primary health care (PHC) approach to address the quadruple burden of disease. However, there is a general lack of leadership in the public sector to ensure that planning, structures, processes and data systems are in place for the health workforce. As a result, the health workforce is ‘unmanaged’ and characterised by attrition (estimated at an annual rate of 25%), shortages and dissatisfaction despite recent efforts to increase salaries. Despite population increases, there has been stagnant to negative growth in posts in this sector [6].

The PHC approach prioritises a preventative approach which is aligned with the branch of medicine, Public Health Medicine, which is concerned with improving the health of the population rather than treatment of individuals. However, there is an acute shortage of these specialists in SA and only about seven qualify annually and the 107 possible training posts (referred to as registrar posts) are only half filled. The shortfall of these medical specialists is partially addressed through the training of public health professionals through various Master of Public Health programmes [6].

The quadruple burden of disease, the PHC approach within the public sector, the overall shortage of a health workforce in the public sector and a shortage of Public Health Medicine specialists necessitate that all health science graduates in SA are ‘new health professionals’. This professional is ‘one who, consistent with their clinical role, encourages and participates in health-producing actions (rather than only providing care) at the individual and population level’ [8]. It is vital that all doctors and other health professionals will be able to ‘recognize and incorporate the concepts of public and population health into their own practice’ [9].

### Public health in the undergraduate medical curriculum in SA

Given the current and predicted public health workforce shortages, it is crucial that health science students are interested in, and prepared for, entering that workforce. Undergraduate public health education is considered crucial for training of the public health workforce to address the existing and emerging public health problems in low- and medium-income countries [10, 11].

As the South African medical education system is predominantly based on the British system, entry to medical school is directly after school and all medical schools are located in public universities [12]. As a result, medical studies are undergraduate studies unlike some other systems such as the American system. Only one of the eight medical schools in SA has a parallel graduate-entry and school-entry medical programme. The medical degree is a 6-year programme: three preclinical years followed by three clinical years, although early clinical exposure is common. This 6-year programme is followed by an internship year and a community service year before graduates are allowed unrestricted (public or private sector) medical practice. Graduates can continue to specialise if interested and if training posts are available in the public sector [12].

In SA, the Health Professions Act 56 of 1974 clearly positions the inclusion of public health in the medical curriculum: ‘Medical public health as a theme shall figure prominently throughout the curriculum’ [13]. However, interpretation of this inclusion is left to the discretion of each medical school and there is no standardisation with regard to the curriculum content, instructional design or allocated time. Another barrier to engage medical students in public health is that they are less interested in healthy people than ill patients and concepts such as populations or public health are so far removed from their individual-patient focus that they are ‘over the conceptual horizon and out of sight for most’ [14].

A lack of interest while studying is a risk as early interest in specialist subject areas is a predictor of future training choices [15]. Health science students are also seldom aware of the possible career choices in the public health field.

In Canada, one of the strategies that have been used to combat the disinterest in public health is the creation of special interest groups (SIGs). These SIGs are used to raise the profile and interest in public health among medical students [16]. These are voluntary, extra-curricular groups that aim to ‘1) provide students with information about the importance of incorporating population and public health concepts into all areas of practice, 2) expose students both to community activities that demonstrate public health concepts and to professionals in the field of public health, 3) provide an opportunity for students to learn, to network, and to develop leadership skills, and 4) provide an opportunity for students to explore public health and preventive medicine as a career option’ [9].

**Fig. 1:**
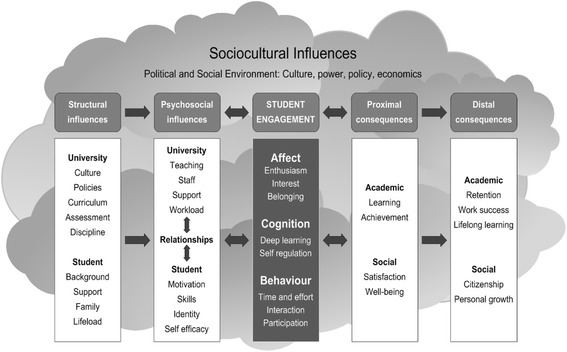
Conceptual framework of engagement, antecedents and consequences [17]

Little has been written on the creation, use and institutional support of these public health SIGs outside of Canada; however, there are numerous other studies that report on the use of SIGs in other specialist areas. A common thread among the literature on SIGs is the desire to engage students. Kahu’s conceptual framework of engagement, antecedents and consequences is therefore useful to understand the various SIG-related studies (Fig. 1) [17].

Kahu proposes her student engagement framework as a ‘psycho-social process, influenced by institutional and personal factors, and embedded within a wider social context’. The framework includes the antecedents and consequences of student engagement as well as indicating the primary direction of influence [17].

Several studies in the health professions argue for the creation of SIGs as an extra-curricular opportunity as their particular field has limited time in the curriculum [5, 18–21]. This argument can be located as a structural influence antecedent in the model as the curriculum constraint stimulated the need for the SIG. In some cases, the SIG was created with a wish to stimulate interest in electives, often research electives, which could be viewed as a structural influence antecedent to strengthen the institutional culture and educational opportunities to develop research skills [19–22].

One study was explicit about the use of SIGs as an opportunity to expose students to role models within their discipline [23]. This identity formation is a psychosocial influence antecedent in student engagement and can be a powerful SIG outcome of linking students and staff.

The majority of studies of SIGs for health science students concentrate on the proximal and distal consequences of student engagement and focus on skills building and clinical exposure [5, 19, 20, 22–25], student advocacy [5, 23], building a professional network [5, 19, 22], service in the community [5, 19] and career choice [5, 19, 20, 23, 25–27]. SIGs are widely considered as a strategy to improve student learning and achievement while contributing to student satisfaction in the short term and ensuring work success (either through clinical competence or through career choice) and citizenship in the long term. In most cases, the SIGs were created with multiple purposes in mind and established within single institutions. However, there are examples of SIGs being established on a national level and financially supported [5, 23]. The public health SIGs in Canada are a prime example of a national initiative that was financially supported and accompanied by a guide on how to create and maintain public health SIGs [9].

While SIGs can be created by students themselves, it is unlikely to happen in public health as health science students generally do not readily see the use of public health in their own disciplines. Creating such a group is then dependent on those who are already qualified or interested in public health and who understand the necessity of exploring strategies to improve interest in public health in this group.

The aim of this case study is to describe the creation and effects of a SIG in public health at one African university.

## Case presentation

An action research study design was used to create and study the SIG. Action research is a method used for innovating scholarly practice; it is participative and collaborative and undertaken by individuals with a common purpose [28].

Action research involves a number of spirals of cycles of:

- Planning a change;
- Acting and observing the process and consequences of the change;
- Reflecting on these processes and consequences and then re-planning;
- Acting and observing; and
- Reflecting [28].

### Creating and studying the SIG

The SIG in public health was created in February 2016 and was maintained throughout the year at the Faculty of Health Sciences at the University of Pretoria, which has a school-entry system. Interested undergraduate health science students were recruited via marketing (flyers and posters) throughout the Faculty of Health Sciences, by word-of-mouth including marketing by two members of academic staff in their lectures.

All interested undergraduate health science students were invited to participate in the SIG. Members of the SIG were also invited to be participants in this case study if they were older than 18 years (the minimum age able to give consent for the collection of data). Participation in the various data collection activities varied according to the availability and consent from the students. The sample sizes in the different cycles of this study varied from 10 to 55 participants.

Paper-based questionnaires were handed out to the participating students at the first meeting to determine a baseline of their knowledge of public health, and an online survey was conducted mid-year. Both surveys explored participants’ perceptions and views regarding public health, the possibility of exploring public health as a career option as well as if they would consider inviting their fellow students to join the SIG. The final activity of the SIG had paper-based questionnaires which were handed out to the participants. All questionnaires were developed based on the literature and were checked for clarity of the questions.

A reflective journal and an audit trail were kept by the researcher throughout the duration of the study.

The trustworthiness of the data was ensured by several strategies to meet the four quality criteria in qualitative research: credibility, transferability, dependability and confirmability. Credibility was obtained through prolonged engagement with the students, persistent observation and triangulation. Triangulation in this study was obtained through writing field notes and using multiple data sources and an audit trail. Transferability was obtained through thick description of data in context and purposive sampling. Dependability was ensured by data saturation. After seven action research cycles, data saturation was reached. Confirmability was ensured by the maintenance of an audit trail.

Hard copies and electronic copies of the data were kept by the researcher. Data analysis occurred concurrently with the data collection strategies. The quantitative data are reported using proportions. The qualitative data from the open-ended questions were analysed by hand using open coding. Results from the online questionnaire were analysed by the software.

### Activities of the SIG

Seven SIG meetings were held during the year (Table 1). Each meeting was also an action research cycle with plan, act, observe and reflect.

**Table 1:** Details of special interest group meetings, action research cycles, data collection strategies and response rates

Cycle	Meeting date	Number of attendees	Response rate	Topic	Tools used for data collection	Facilitator
1	28/02	16	100%	Introduction to the SIG	Paper-based questionnaire reflections	Researcher
2	23/03	10	N/A	Feedback on questionnaires, way forward	Researcher’s reflections	Researcher
3	22/04	7	N/A	Discussion on public health	Researcher’s reflections	Researcher Public Health Medicine consultant
4	03/05	7	N/A	Outbreak scenario session 1	Researcher’s notes	Guest speaker
5	10/05	5	44%	Outbreak scenario session 2	Online questionnaire researcher’s reflections	Guest speaker
6	19/07	8	N/A	Planning for the PH seminar, discussion on relevant topics	Researcher’s reflections	Researcher
7	24/09	56	98%	Seminar on Gender-Based Violence: An Approach to Forensic Evidence	Paper-based questionnaire researcher’s notes reflections	Foundation For Professional Development

*N/A* not available

### SIG outcomes

A total of seven sessions were held culminating in a seminar. The first six SIGs were held in a private room, and the seminar was held in a lecture hall. The first SIG was a welcoming session that explained the goals of the SIG. This session also explored what participants hoped to achieve as well as suitable meeting days and times.

In the second session, the researcher provided feedback on the questionnaires, as well as discussion about the interests and next activities for the SIG. Participants were keen to do more experiential learning via field trips. Participants generated two topics that were of interest to them: the envisaged national health insurance and gender-based violence, both of which were prominent in the popular press at the time. Finally, participants were interested in employment possibilities in public health. As a result, the third session focussed on the scope of public health, employment and study options and was led by a Public Health Medicine consultant.

The fourth and fifth sessions were outbreak scenario sessions. The topic was the only topic generated by the researcher as a practical example as baseline results showed participants had limited understanding about public health. These sessions were led by a guest lecturer who had been involved in the latest Ebola outbreak, and a case study from the Centers for Disease Control and Prevention was used. Refreshments were served as part of the evening events.

The sixth session involved the planning for the public health seminar, possible partners, dates and marketing as well as the details about what the topics for the seminar would be.

The seventh session was a seminar on Gender-Based Violence: An Approach to Forensic Evidence. The participants attended the session on a Saturday as the seminar was a full-day event. Refreshments and lunch were provided by the presenters of the seminar.

### SIG participants

The composition of the SIG fluctuated. The core group of 16 students consisted of 12 females (75%) and 4 males (25%). Despite faculty-wide marketing, all the participants were medical students (second to fifth years). A total of four second-year students (25%), five third-year students (31%), two fourth-year students (12%) and five fifth-year students (31%) attended the scheduled sessions.

### Marketing of the SIG

The participants heard about the SIG from several sources, via WhatsApp (19%, *n* = 3), Facebook (25%, *n* = 4) and academic staff (31%, *n* = 5). Only one student (6%) knew about the SIG via the posters that had been posted widely in all the Faculty buildings. Three participants heard from other students (19%).

### Motivation for involvement

At baseline, a total of 12 participants (75%) said their reason for getting involved was to learn more about public health. Two participants (12.5%) thought that they would like to pursue public health as a possible career choice, and six (38%) wanted to gain experience in public health. Few (19.8%, *n* = 3) wanted to contribute to improving the health of the population. A total of 11 participants gave a single reason for joining, and five listed multiple reasons for joining. An example by one participant was ‘I like to explore any new ideas and opportunities. This seemed to be a good idea to learn more about PH and everything this entails’.

Participants’ aspirations for participation in the SIG ranged from gaining knowledge and experience (50%, *n* = 8), understanding public health (31%, *n* = 5), participating in research (25%, *n* = 4), going on field trips (12%, *n* = 2) and using the SIG as a means of networking (25%, *n* = 4).

### Preferred meeting times and communication

Despite the range of participants being over 4-year groups with varying schedules and commitments, Tuesdays between 1700 and 1745 hours were identified as the most convenient meeting time. On average, the SIG meetings lasted between 45 and 60 min while the two outbreak scenarios lasted between 60 and 90 min. The seminar was a full-day event that took place on a Saturday. All the participants preferred WhatsApp as a method of communication.

### Emerging interests and student leadership

All the participants were interested in attending sessions similar to the outbreak control investigation in future and reported that they were more interested in public health compared to the beginning of the year. When asked if they would recruit fellow students, six of the seven participants responded positively (86%), and the remaining person was unsure.

At this mid-year point, six (38% of the original 16) responded that they would consider public health as a possible career choice in future. These six students turned out to be the six key players in the SIG.

### Expanding membership

During the SIGs, a decision was made to market the SIG and recruit new members via a seminar. During these discussions, the topic—Gender-Based Violence: Approach to Forensic Medicine—was decided on as it was a suggestion by one of the key players. The identification of an external training partner—the Foundation for Professional Development (FPD)—was facilitated by a member of the academic staff. The success of the seminar was dependent on the six core group members who facilitated the marketing and communication between the university and the organisers for the arrangements. The original arrangement for the use of university facilities was done by the researcher and then handed over to the students. The seminar was an open session with participants from different health science fields.

Six seminar participants (11%) were a combination of lecturers, doctors, radiographers and unit managers at an emergency centre. Among the remaining 50 participants, 34 were medical students (68%), 10 nursing students (20%), four psychology students (8%), one optometry student (2%) and one clinical associate student (2%).

### Marketing of the seminar

The marketing of the seminar was done by putting up posters at the university, by word-of-mouth and posted on Facebook. The poster was designed by the SIG members, and the SIG members, researcher and two members of the academic staff were all involved in the marketing of the seminar. Participants were asked how they had heard about the seminar (Table 2).

**Table 2:** Methods of marketing for the seminar

Method	Number of respondents	Percentage
SIG members	10	17.85
Facebook	10	17.85
Nursing department	7	12.5
WhatsApp	7	12.5
Email	6	10.71
Other students	5	8.92
Supervisors	3	5.35
Public forum	2	3.58
Work	2	3.58
FPD	2	3.58
Posters at faculty	1	1.79
Offsite posters	1	1.79
Total	56	100%

### Motivation for participation in the seminar

A total of 22 of the participants (39%) attended the seminar because they found it is an interesting subject and of personal interest: ‘This is of personal interest to me as this topic has directly to do with my studies’. Nineteen (34%) wanted to gain knowledge of the subject, and four (7%) said the topic is of interest to their career and future studies: ‘I would like to know more about this topic and what it is about, I feel it is important for my career in future’.

### Awareness about the SIG and interest to join

The responses of the lecturers, unit managers and professionals were removed for analysis. Of the remaining 50 participants, five were already a part of the SIG and were removed. Out of 45 participants, 21 (46.7%) were aware of the SIG. A total of 37 participants (82.2%) said that they would be interested in joining the SIG next year, while eight (17.8%) were not interested.

## Discussion

This case study describes the creation and effects of a SIG in public health at one African university. The rationale for the creation of the SIG was rooted in the growing concern regarding the country’s health workforce shortages in the public sector and the need that health science graduates need to be ‘new health professionals’ who can recognise and apply the concepts of public and population health in any sector or setting [8].

The constraint in curriculum time acted as structural influence antecedent for the creation of this SIG. A second antecedent to student engagement was the possibility to provide suitable role models in public health.

This case study highlights the challenge of competing interests and retaining student engagement. At the start of the study, a total of 16 students attended the first meeting but the number at the scheduled meetings decreased over time. This decrease mirrored the increase in pressure due to their studies and extra-curricular activities.

Despite marketing being done throughout the entire Faculty, only medical students showed an interest in joining the public health SIG. This finding confirms what has already been described in Canada where only the medical schools had active public health interest groups [5]. This finding could be as a result of the most successful marketing strategy in this study, which was the academic staff who do not lecture to other health science students. It could be argued that the reason that this marketing strategy was more successful than any other is related to role modelling and identity formation, which was not the case for the other health science students.

The topics and activities suggested by the participants and the academic staff were stimulated by what was in the popular press at the time and were therefore topics that favoured student interest and engagement. Experiential learning opportunities, such as field trips and involvement with communities, proved difficult in this first year due to the restricted common time available to all SIG members. Also, the number of sessions was less than planned due to security concerns as a result of national student protests about language and fees.

Despite these restrictions, it was still possible for the SIG to meet the objectives of raising awareness and understanding of public health. Raising awareness and understanding of public health are proximal consequences of student engagement within a SIG, while a possible distal consequence was the interest in public health as a possible career choice.

There were six key players who attended every session and who took leadership in the arrangements of the seminar. The emergence of this core group appears to be critical in the first step in the SIG becoming student-led and internally driven, consistent with the Canadian experience [5]. The motivation of this core group was not examined in the study, but might be related to the social satisfaction, personal growth and citizenship that Kahu lists as consequences of student engagement [17]. The continuation of the SIG is dependent on the core group. It is clear that recruitment drives such as the seminar are vital in raising visibility of public health and the SIG.

## Conclusion

The quadruple burden of disease and the health workforce shortages in the public sector imply that every graduating health professional must be able to contribute to public and population health, irrespective of their setting. This SIG was an important and relevant strategy to increase public health interest among some medical students at the University of Pretoria. The SIG provided a useful vehicle to address some of the antecedents to student engagement, namely curricular constraints and identity formation. Positive outcomes of the SIG were the proximal consequence of learning and the possible distal consequence of career choice.

Ideally, any public health SIG should be student-led to be sustainable. However, a preparatory period that is characterised by a high level of involvement by those already qualified or interested in the discipline is needed. This preparatory period is the incubator for the development of ideas and allows a core group of interested and committed students to emerge. Instead of the traditional marketing strategies, high visibility activities such as seminars presented on topics that align with students’ clinical interests appear to be more successful in recruiting new members.

This case study is limited in that it was done in a single setting and had student protests and security concerns which limited the planned number of meetings and the possibility to obtain richer results. This case study did not explore the reasons why those who said they were interested ultimately did not attend, nor did it explore the motivations of the core group of students. Other studies should explore these two aspects, and a longitudinal study to follow-up on actual career choices is suggested.

The insights gained from this case study are important and relevant for public health educators who wish to use SIGs as a strategy to stimulate interest in public health among health science students. These students will be adding to the limited and overworked workforce of the country. But, having graduates with a wider population-level perspective of the health system and its challenges, as well as strategies on how to navigate it successfully, could be gained from exposure to public health SIGs. The creation of networks to peers and public health professionals from such SIGs is immeasurable. The lessons learnt from this case study can be used to create and sustain public health SIGs in other contexts.

## Abbreviations

FPD: Foundation for Professional Development
SIG: Special interest group
